# Exploring Advancements in the Treatment of Amyotrophic Lateral Sclerosis: A Comprehensive Review of Current Modalities and Future Prospects

**DOI:** 10.7759/cureus.45489

**Published:** 2023-09-18

**Authors:** Pranvera Hoxhaj, Natasha Hastings, Meet Popatbhai Kachhadia, Riya Gupta, Udeept Sindhu, Shreya A Durve, Areeba Azam, María J Auz Vinueza, Bhuvan, Shwe H Win, Deepak C Rathod, Aiman P Afsar

**Affiliations:** 1 Medicine, University of Medicine, Tirana, Tirana, ALB; 2 Obstetrics and Gynaecology, Scher & Kerenyi MDS, New York, USA; 3 Medicine, St. George's University School of Medicine, St. George's, GRD; 4 Internal Medicine, Pandit Dindayal Upadhyay (PDU) Medical College, Civil Hospital Campus, Rajkot, IND; 5 Medicine and Surgery, Shri Atal Bihari Vajpayee Medical College and Research Institute, Bangalore, IND; 6 Medicine and Surgery, Kasturba Medical College, Manipal, Manipal, IND; 7 General Medicine, Sri Ramachandra Institute of Higher Education and Research, Chennai, IND; 8 Medicine, Lahore Medical and Dental College, Lahore, PAK; 9 Critical Care, Hospital de Especialidades de las Fuerzas Armadas N1, Quito, ECU; 10 Internal Medicine, Government Medical College, Amritsar, Amritsar, IND; 11 Medicine, University of Medicine, Magway, Magway, MMR; 12 Medicine, Chandramma Dayanand Sagar Institute of Medical Education and Research, Harohalli, IND; 13 Medicine, Maulana Azad Medical College, New Delhi, IND

**Keywords:** supportive care, robotics, pharmaceutical interventions, psychological support, cell therapy, gene therapy, neurodegenerative, amyotrophic lateral sclerosis

## Abstract

Amyotrophic lateral sclerosis (ALS) is a fatal and incurable disease requiring a multidisciplinary treatment approach and a collaborative therapeutic effort. A combination of both upper and lower motor neuron degeneration ultimately leads to respiratory failure, similar to other dementia-type neurodegenerative diseases. The aim of this paper is to pioneer current ALS research by carrying out a narrative literature review of the current treatment modalities of the disease. Through these efforts, we hope to condense the most pertinent information regarding current treatments and enhance the management of ALS patients as a whole, giving these patients a better quality of life as the search for a cure continues. We used a Pubmed search strategy and specific MeSH terms for the selection of the literature articles using the keywords "ALS," "new treatment," "treatment," and "symptomatic treatment." A combination of pharmaceutical interventions, psychological support, and physical rehabilitation has been most effective in enhancing the quality of life of patients with ALS (PALS). Among potential pharmacological therapies, only a few have been approved by the US Food and Drug Administration(FDA) to be used to treat ALS and its symptoms. Other treatment modalities being considered include gene therapy, cellular therapy, psychological therapy, physical therapy, and speech therapy, alongside robotics, alternative feeding methods, and communication devices.

## Introduction and background

Amyotrophic lateral sclerosis (ALS) is a rare neurological disease that affects motor neurons in the central nervous system, resulting in progressive degeneration and eventual death of these electrochemical connections. ALS causes a combination of upper and lower motor neuron disease, and the symptoms vary depending on the muscle controlled by the affected neurons and whether upper or lower motor neurons are predominantly affected. The main manifestations of upper motor neuron disease are muscle weakness, increased muscle tone and stiffness (spasticity), increased reflexes (hyperreflexia), and abnormal speech and swallowing [1]. In contrast, manifestations of lower motor neuron disease present as muscle wasting (atrophy) and twitching (fasciculations), loss of muscle tone (flaccid paralysis), and decreased reflexes (hyporeflexia). The exact cause of sporadic ALS is unknown. It has been hypothesized that dysfunctional and interconnected molecular mechanisms contribute to disease progression in ALS. Gene mutations, most commonly *C9orf72, SOD1, TDP-43, FUS,* and *TBK-1*, account for 15% of all patients with ALS (PALS). Of these mutations, the expansion that occurs in the *C9orf72 gene *accounts for 30-50% of familial ALS and 7% of sporadic ALS [2]. Affecting approximately 4.42 per 100,000 individuals, death from the illness usually occurs due to respiratory failure three to five years after the onset of symptoms. Males are slightly more affected by the disease than females [1]. Due to the inevitable fatal nature of this disease and its increasing incidence, resources have been allocated toward discovering treatment modalities that will positively affect patient outcomes and slow disease progression. Effective and safe treatment modalities that may change the progression of the disease remain elusive, with current management protocols used in clinical practice showing variable results in improving morbidity and mortality [3]. The diagnosis of ALS is suggested by clinical examination that reveals both upper and lower motor neuron failure. Electrodiagnosis analysis forms a crucial part of the diagnosis. A combination of both physical exams and clinical diagnostics is necessary for correctly diagnosing ALS. Conventional imaging studies, such as magnetic resonance imaging (MRI) and computed tomography (CT) scans, are used to exclude other diseases that present similarly to ALS, demonstrating high levels of sensitivity [4,5]. Cellular therapy has shown substantial therapeutic potential in treating PALS over recent years. Still, the safety and efficacy of cellular therapy in PALS remain under debate [1]. Cellular therapies are working to optimize the dose required for treatment and to find an optimal dosing method that may further encourage full adherence to treatment by PALS. Another crucial component of the multidisciplinary approach to ALS management is physical therapy (PT). PT can address problematic symptoms of ALS, such as fatigue and muscle stiffness, through energy conservation and stretching exercises, respectively [3]. Restrengthening key respiratory muscle groups is undeniably necessary to delay fatality due to respiratory failure. PT has been shown to significantly improve the quality of life of PALS [6]. PALS experience a range of physical, emotional, and cognitive challenges, which can make it difficult to address all aspects of their care. Developing interventions that are tailored to treat the cognitive and behavioral side effects of ALS is complex. Further research is needed to develop nonpharmacological treatments that may reduce the psychological burden that comes with the disease for both PALS patients and their caregivers [7].

This narrative review synthesizes a wide range of research and expert opinions on ALS and its treatments into a concise and comprehensive overview, which includes pharmacological therapies, PT, respiratory support, and palliative care approaches. Overall, through this review, we aim to develop a vital reference tool capable of enhancing the understanding of ALS and aiding in the improvement of treatment outcomes.

## Review

Methodology

**Figure 1:**
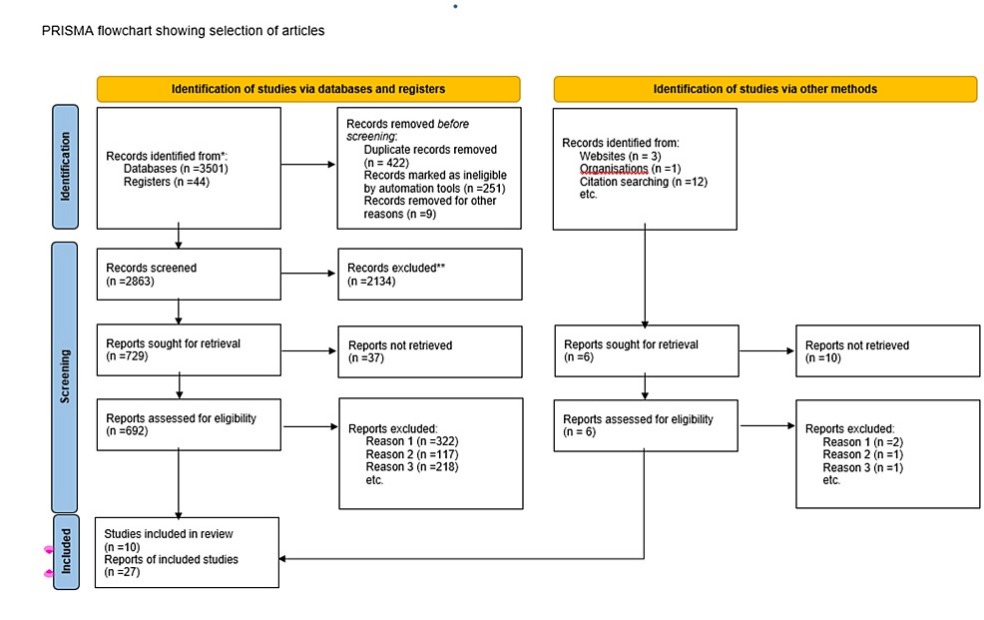
PRISMA Flowchart Showing the Selection of Articles

This is a narrative literature review of ALS and current treatment modalities of the disease based on articles obtained from PubMed using the keywords “ALS," "new treatment," "treatment," and "symptomatic treatment.” Using the search strategy involving "ALS diagnosis, diagnostic imaging, prevention and control, psychology, radiotherapy, rehabilitation, surgery, and therapy" as MeSH terms for the selection of the literature articles, we found 786 articles. Inclusion criteria involved primary research publications, systematic reviews, and meta-analyses published in English, focusing on the treatment of ALS in humans from the timeline spanning from 2015 to July 2023. Animal studies and studies without relevant outcomes were excluded. The titles and abstracts of the retrieved articles were screened independently by three reviewers to determine their relevance to the research topic and were narrowed down to 37 articles (Figure 1). Full-text articles were further evaluated in detail. This narrative review has been done considering the current situation of PALS to determine the latest advancements in its treatment and their effectiveness compared to the conventional therapies available. This could have an impact on disease progression and thus the ability to influence the severity of possible complications.

Results

Although ALS remains incurable, effective management of symptoms and proactively addressing current clinical complications hold great significance for the patients affected by the disease. Fortunately, there is strong evidence suggesting that early detection of the disease contributes to extending patient survival. Given the heterogeneity of symptoms of ALS, supportive and palliative care can be provided by specialized multidisciplinary teams, composed of healthcare professionals providing care and support for the patients and their families. Our research reveals that treatments for PALS predominantly fall into two distinct categories. The first category includes pharmaceutical compounds that mainly influence disease progression, and the second category includes supportive and palliative care of the symptoms.

Riluzole, the original US Food and Drug Administration (FDA)-approved remedy for ALS, belongs to the benzothiazole class of drugs and acts as a glutamate antagonist that contributes to the death of upper and lower motor neurons in this disease. While riluzole can slow down the disease’s progression and impact muscle function deterioration, it only results in a minor increase in survival, spanning from three to six months [8,9]. Edaravone, the next FDA-approved medication for the treatment of ALS, works as a powerful antioxidant and scavenger of free radicals. While the exact way in which edaravone works to treat ALS is not fully understood, its antioxidant properties are thought to play a crucial role, as oxidative stress is a contributing factor to disease progression in PALS [10,11]. Sodium phenylbutyrate/taurursodiol is a medication approved for treating PALS because it reduces cellular stress pathways and promotes nerve cell survival. Patients treated with this medication have shown a slower decline in daily functioning and have longer overall survival rates compared with PALS treated with a placebo [12]. Tofersen is an approved RNA-based therapy designed to reduce the synthesis of superoxide dismutase type 1 (SOD1) protein by degrading toxic clumps of SOD1 mRNA (superoxide dismutase type-1 messenger ribonucleic acid). Tofersen initiation reduced neurofilament-axonal (nerve) injury and neurodegeneration and improved disease outcomes [13-15]. The dextromethorphan hydrobromide/quinidine sulfate (DMQ) combination is a medication that is prescribed in patients with ALS to treat the underlying pseudobulbar affect (PBA). This treatment has been shown to improve three bulbar functions: swallowing, speech, and salivation. According to a trial study, both PALS groups treated with DMQ, regardless of having or not having PBA symptoms, showed impactful improvements in their total Center for Neurologic Study - Bulbar Function Scale (CNS-BFS) scores [16,17].

Some common therapy interventions that improve respiratory outcome measures and increase survival of PALS are cough augmentation, lung volume recruitment training, airway clearance techniques, assisted breath stacking, and manually assisted cough [18,19]. Dysphagia management and nutritional support involve detecting the evaluation of swallowing by fibro-endoscopy and fluoroscopy, changes in diet consistency, the loss of body weight, and indicating the start of alternative feeding methods such as percutaneous endoscopic gastrostomy (PEG) or jejunostomy [20]. Speech therapy is indicated in the initial stages. In advanced settings, speech therapy is accompanied by an electronic communication device (such as computers, telephones, voice output, and keyboard or head-eye-tracking devices) [21-23]. PT is an integral component of the ALS multidisciplinary team and is tailored to the individual’s needs and goals. Focusing on addressing the symptoms and maximizing function and participation can enable PALS to achieve a better quality of life. The most commonly used PT techniques for PALS are a range of motion (ROM), stretching, resistance, and aerobic exercises [6,24]. Psychological distress is a common experience for PALS, often leading to feelings such as anxiety, hopelessness, despair, demoralization, and depression. Effective psychological interventions should be used to assist the patient and their loved ones in managing the fear of death and dying and dealing with the challenges that arise due to visible changes in relationships and social roles. Table 1 presents the symptom-wise pharmacological treatment of ALS.

**Table 1:** Symptom-Wise Pharmacological Treatment of ALS

Indication	Medication	Dose	Side Effects
Spasticity	Baclofen	5mg 1-3 times/day	Hypotonia, drowsiness, nausea, vomiting, excessive sweating
	Nabiximols	Buccal spray: 4-8 sprays/day	Tachycardia, dizziness
	Tizanidine	2-4mg 2 times/day	Hypotension, dizziness, asthenia, xerostomia
	Onabotilinum- toxin A	Different units of intramuscular site injection	Xerostomia, dry eyes, dropping eyebrows, headache, muscle weakness
Cramps/Pain	Tizanidine	2-4mg 2 times/day	Hypotension, dizziness, asthenia, xerostomia
	Gabapentin	Titrating from 100mg/day to 900mg 3 times/day orally	Fatigue, arm and foot edema, mood changes, vomiting, blurred vision
	Levetiracetam	500-1000mg twice/day orally	Fatigue, anxiety, headache, weight loss, sleepiness
	Mexiletine	150mg twice/daily orally, starting dose 150mg/day for 3 days	Dizziness, hepatotoxicity, ataxia, nausea, vomiting
	Baclofen	5-10mg 3 times/day up to 80-120mg/day orally	Drowsiness, hypotonia
	Quinine sulphate	260-325mg daily	Abdominal pain, chills, tinnitus, impaired hearing
	Phenytoin	300mg daily orally	Headaches, drowsiness, vomiting
	Vitamin E	400 UI (unite) /day orally	
Fatigue	Modafinil	100-200mg/day in 1-2 doses orally	Headache
Sialorrhea	N-acetylcysteine	200-400mg 3 times/day orally	Rash, bronchospasms, chest tightness
	Scopolamine	1 transdermal patch every 3 days	Tachycardia, xerostomia, hyperthermia, flushed skin, urinary retention, constipation
	Hyoscyamine	1-2 transdermal patches every 3 days, OR oral sustained release 0.375-0.75mg every 12 hours OR fast-acting 0.125-0.25mg before meals	Tachycardia, xerostomia, hyperthermia, flushed skin, urinary retention, constipation
	Atropine	0.4mg every 4-6 hours as sublingual drops	Tachycardia, xerostomia, hyperthermia, flushed skin, urinary retention, constipation
	Glycopyrrolate	1-2mg 3 times/day orally	Xerostomia, nasal congestion, flushing, constipation, nausea, vomiting
	Rimabotulinum- toxin B	Parotid gland injection of 500-1500 units, submandibular gland injection of 250 units in each	Dysphagia, xerostomia, antibody formation
Pseudobulbar affect	Amitriptyline	Titration from 10-25mg/day orally to 10-150mg/day	Sedation, suicidal thoughts and behavior
	Fluvoxamine	100-200mg/day orally	Insomnia, headache, drowsiness, nausea, suicidal thoughts, and behavior
	Dextromethorphan quinidine	20 and 10mg/day for 7 days, then twice/day orally	Diarrhea, nausea, weakness
Anxiety	Buspirone	10mg 3times/day orally	Dizziness, headache, nausea, trouble sleeping
	Diazepam	2-10mg 3 times/day orally	Drowsiness, headache, constipation,
	Lorazepam	0.5-1.0mg 2 times/day orally	
	Mirtazapine	5-30 mg	Drowsiness, constipation, headaches
Depression	SSRI antidepressants	20-100mg orally daily	
	Tricyclic antidepressants	20-100mg dailly orally	
	Bupropion	100mg 2 times/day orally	
	Venlafaxine	37.5-75mg 2 or 3 times/day orally	
Urinary urgency	Oxybutynin	2.5-5mg 2 times/day orally OR 3.9mg transdermal patches	Headache, dry eyes, vomiting, stomachache, vertigo
	Tolterodine	1-2mg 2 times/day orally	Vertigo, headache, xerostomia

Discussion

ALS is an overwhelming neurodegenerative disease that affects patients physically and psychologically, reducing their quality of life and survival. Currently, there is no available cure for ALS. Scarcely any drugs have been approved by the FDA that can slow the course of the disease and improve the quality of life. Therefore, the management of ALS remains supportive and symptom-based. In recent years, research on new treatment strategies has increased, taking heed of gene therapy, cellular therapy, and neuroprotective agents.

Pharmacological management

There are limited approved drugs that slow disease progression by prolonging autonomy and increasing survival rates (measured by the ALS functional rating scale (ALSFRS-R) [3].

Riluzole, (1,6-(Trifluoromethoxy)-2-Benzothiazolamine)

A benzothiazole derivative was the first drug approved by the FDA for the treatment of ALS and has been used since 1995, showing a potentially beneficial effect for patients. Clinical studies have shown that riluzole treatment reduces the loss of motoneurons and increases survival by 9% in one year. This drug acts by multiple mechanisms to decrease excitotoxicity. It is thought that riluzole may have some neuroprotective effects. This is because its anti-glutamatergic agent inhibits the presynaptic release of neurotransmitter glutamate by acting as a specific sodium channel blocker on presynaptic neurons. In addition, it reduces the reuptake of gamma-aminobutyric acid (GABA) and enhances GABA receptors. Recent studies have associated riluzole with another mechanism of action (MOA). It inhibits protein kinase CK1δ catalytic activity, which avoids the cytoplasmic compartmentalization of TDP-43 (transactive response DNA binding protein of 43 k), resulting in the suppression of excitotoxicity. The recommended initial dose of riluzole for the treatment of ALS is 50 mg taken orally twice daily. After initiation of therapy, the dose can be increased to a maximum of 100 mg twice daily based on individual tolerance and clinical response. It is considered a well-tolerated drug among patients, even though it has shown some adverse effects, such as gastrointestinal symptoms, dizziness, and asthenia [1,3,25].

Edaravone (MCI-186)

Twenty-two years later, in 2017, edaravone was the second compound to be FDA-approved for the treatment of ALS. It is a free radical scavenger that protects motor neurons from damage by free radicals and oxidative stress by eliminating lipid peroxyl radicals and peroxy nitrate [1]. There have been multiple trials that have studied the efficacy of this drug. A randomized, double-blind, placebo-controlled phase III study with 137 participants proved the use of edaravone as an ALS treatment. It included only patients with early-stage ALS, defined as a disease duration of two years or less, a score of at least two for every item of the revised amyotrophic lateral sclerosis functional rating scale (ALSFRS-R), and forced vital capacity (FVC) of 80% or more, along with a probable or definite ALS diagnosis defined by the El Escorial criteria [3]. The study demonstrated a decrease in motor decline and motor neuron degeneration. Furthermore, edaravone efficacy has been demonstrated by its antioxidant properties and the ability to reduce mutant SOD1 accumulation. Edaravone’s chemical name is 3-methyl-1-phenyl-2-pyrazolin-5-one, and it is available as an intravenous (IV) infusion containing 30 mg edaravone in 100 mL of isotonic solution. The recommended dose of the initial treatment cycle with edaravone is 60 mg administered via 60-minute IV infusion once daily for 14 days, followed by a 14-day drug-free period. Subsequent treatment cycles consist of once-daily dosing for 10 of 14 days, each continued by a 14-day drug-free period. The drug is 92% protein-bound, primarily to albumin. The drug is metabolized in the liver and kidney. It is excreted mainly in the urine in its glucuronide conjugate form. Clinical trials have demonstrated that, even though edaravone has many side effects, it is a treatment that is well tolerated and, more importantly, decreases disease progression. Common adverse events include contusion, confusion, constipation, eczema, headache, bruising, gait disturbance, and contact dermatitis. Severe side effects were also present, such as hypersensitivity, dysphagia, sulfite allergic reactions, and anaphylaxis [10,11,26].

Sodium Phenylbutyrate and Taurursodiol Combination (Relyvrio)

The combination of these drugs has exhibited an effect in reducing neuronal cell death and oxidative injury by diminishing stress in the endoplasmic reticulum (ER) and mitochondrial dysfunction. A reduction in disease progression has been documented with the use of both drugs. Sodium phenylbutyrate is an inhibitor of the histone deacetylase enzyme, which regulates transcription and allows normal protein aggregation. Studies in animal models showed that sodium phenylbutyrate improved cell survival. Moreover, clinical trials have demonstrated that this drug increases blood histone acetylation levels. The efficacy of Relyvrio (sodium phenylbutyrate and taurursodiol) was demonstrated in a 24-week, randomized, placebo-controlled, multicenter, double-blind, parallel-group study. In the trial, 137 PALS received either Relyvrio or a placebo. The study showed that the patients treated with Relyvrio experienced a slower rate of decline in the clinical assessment of daily functioning than those who received the placebo. Additionally, prolonged survival was observed in the long-term analysis of patients who received Relyvrio when compared to the placebo arm. Notable adverse reactions suffered by patients taking Relyvrio include upper respiratory tract infections, abdominal pain, nausea, and diarrhea. Relyvrio contains taurursodiol, a bile acid, which may cause worsening diarrhea in patients with additional disorders that interfere with bile acid metabolism. Taurursodiol is commonly used to treat chronic cholestatic liver diseases and gallstones. Its anti-apoptotic effect occurs through either regulating the expression of specific targets of apoptosis or mitochondrial membrane stabilization. This results in a neuroprotective effect and decreases disease progression. Sodium phenylbutyrate/taurursodiol has been developed as an oral suspension that contains 3 g of sodium phenylbutyrate and 1 g of taurursodiol. The drug was given once daily for three weeks, followed by twice a day [3,12,27,28].

Tofersen

A SOD1 antisense oligonucleotide decreases the synthesis of SOD1 protein. The MOA occurs through the destruction of SOD1 mRNA. Through this mechanism, the drug reduces plasma neurofilament light (NfL), a biomarker of neurodegeneration and axonal injury. In a double-blind, placebo-controlled clinical trial, tofersen reduced the levels of SOD1 proteins in the CSF (cerebrospinal fluid) by 36%. Earlier Tofersen initiation led to better measurement outcomes. These findings show a crucial clinical benefit in PALS. The reduction in NfL was consistent across all subgroups based on disease duration since symptom onset, site of symptoms, and use of other medications for ALS treatment. Patients were administered three initial doses of 100 mg (15 ml) of tofersen intrathecally at intervals of 14 days. After this, the therapy consists of a maintenance dose administered every 28 days. Treatment with torsefen showed moderate side effects: procedural pain, postlumbar syndrome related to lumbar puncture, headache, myalgia, and arthralgia [14,15].

Emerging modalities

Gene Therapy

Encouraging new treatment options for ALS include gene therapies that work by silencing the expression of harmful genes by replacing the mutated gene with the regular copy or editing the mutant genome, adding genes that introduce a protective or favorable factor, or adding genes with a knockout effect targeting RNA to decrease the expression of the causative gene [1].

Cellular Therapy

It is a treatment modality consisting of both human and nonhuman cells that target multiple pathogenic mechanisms: host cell replacement, immunomodulation, neurotrophic factor secretion, and predominantly neuroinflammation. Seven major cell types are used: mesenchymal stem cells (MSCs), embryonic stem cells, mononuclear cells, neural precursor cells, neural stem cells, glial stem cells, and induced pluripotent stem cells (IPSCs). Transplanted cells can be administered via different routes: intrathecal, intravenous, and intramuscular injection [1,2]. The aim is to make use of stem cells in the transplantation process where there is a displacement of dysfunctional motor neurons for the repair of the neuromuscular pathology of ALS.

Experimental Stem Cell Therapies

Stem cells play the most important role in the sustenance and regeneration of tissues in our body. Their exclusive properties include multipotency, self-restoration, and long-term survival. Stem cell therapies have proven to have encouraging results regarding the regeneration and replacement of neural tissues. Somatic cells of ALS patients can be reconfigured into IPSCs, which can generate certain neuronal stem cells. These IPSCs taken from PALS could play a major role in cell transplantation for the treatment of ALS and in understanding the pathophysiology of the disease [10]. NurOwn is a treatment in an experimental phase that uses MSCs, which are engineered to secrete neurotrophic factors that induce nerve regeneration by signaling neuron-neuron communication. It is deduced that injecting MSCs into the spinal canal showed a distinctive improvement in motor neuron survival. Analyses indicated a treatment benefit for patients with less severe ALS at the trial's start [29]. NeuroNata-R (lenzumestrocel) is an MSC-based therapy that uses intrathecal autologous bone marrow-derived MSC injections. The trial study showed significant therapeutic benefits lasting at least six months with safety in PALS [30]. AstroRx is a trial medication that contains nervous system support cells called astrocytes engineered from human embryonic stem cells. Findings from a small phase I/II study suggested that this experimental therapy may help slow ALS progression [31].

Other therapeutic strategies

In the last few years, many therapies have been tested. Among them are axonal transport, posttranslational modification, DNA damage, nucleocytoplasmic transport, and molecular tweezers. Of the strategies mentioned, posttranslational changes (PTMs) are one of the therapies with more studies regarding their use in ALS treatment. PTMs influence proteins that are involved in ALS pathogenesis, such as FUS, SOD1, and TDP-43. Regarding the other therapies, axonal and nucleocytoplasmic transport strategies are currently being tested in clinical trials, with no clear understanding of their MOA. No clinical trials are addressing DNA damage [3].

Robotics

There are striking technological innovations that are effective in the everyday use of ALS patients. Exoskeleton robotic devices are designed to enhance the gait capability of ALS patients. Such electromechanical wearable systems can be monitored via several aspects, such as speed, position, force, actuator, and algorithms, or by engaging electroencephalographic (EEG) signals or electromyographic (EMG) signals. Electric-powered wheelchairs, monitored through electro-optic tools, also proved to be helpful for ALS patients. In patients with restricted motor functions and severe disabilities, electrically adjusted beds can aid them with changing positions. Brain-computer interfaces (BCIs) could also benefit ALS patients with severe disabilities [3].

Symptomatic Treatment

Symptomatic management has been directed to reduce clinical features that affect the quality and expectancy of life. It focuses on treating symptoms such as pain, muscle spasticity, respiratory insufficiency, and mood disturbances. Approximately 75% of patients develop limb weakness, and 25% develop bulbar symptoms. As mentioned above, bulbar symptoms are characterized by weakness, tongue twitching, dysarthria, and dysphagia, the last two being the most common in ALS [1].

The Nuedexta treatment trial showed that the administration of dextromethorphan and quinidine improved pseudobulbar effects such as speech, swallowing, and salivation. Dextromethorphan is a sigma-1 receptor agonist that enhances serotonin uptake by inhibiting ion channel gates and potentiating ligand-gated channels. On the other hand, quinidine inhibits the cytochrome p450 isoenzyme, protecting dextromethorphan from o-demethylation. This increases the systematic bioavailability of dextromethorphan. At the beginning of the study, it was debatable whether DMQ treatment would improve impaired speech and swallowing in patients who did not exhibit pseudobulbar affect. However, it was found that all PALS, regardless of whether ALS disease was associated with or without PBA, experienced an equivalent improvement in their total CNS-BFS scores after DMQ treatment. Symptomatic frontal disinhibition (pseudobulbar affect), characterized by sudden episodes of crying and/or laughing, can also be treated by dextromethorphan hydrobromide and quinidine sulfate drugs that were approved by the FDA in 2011. The dextromethorphan hydrobromide and quinidine sulfate combination should be given at dosages of 20 mg and 10 mg, respectively [3,23].

Regarding the treatment of other symptoms that are present in ALS patients, spasticity can be treated by cannabinoids, tizanidine, and baclofen, and sialorrhea can be treated by botulin toxin injected into the salivary gland or anticholinergic medication. Medications such as magnesium supplements, quinine sulfate, gabapentin, or carbamazepine are aimed at treating muscle cramps [1].

Other symptoms that are present in ALS patients are treated with a variety of medications. For the treatment of spasticity, tizanidine, baclofen, and cannabinoids are used. For muscle cramps, magnesium supplements, gabapentin, carbamazepine, and quinine sulfate are used. Sialorrhea is treated by injecting botulin toxin into the salivary gland or anticholinergic medication [2].

PT

In ALS, PT has to be considered a palliative and supportive healthcare arbitration. Palliative, symptomatic, and rehabilitative treatments are commonly delivered by an interdisciplinary team that consists of physical therapists, neurologists, nurses, and home healthcare support and can help improve the quality of life and change the end-of-life experience of patients and their families. There are three phases of disease progression in PALS: early (initial), middle, and late (terminal) phases. In the early phase, individuals can independently carry out their activities of daily living (ADLs). In the middle phase, mobility restriction is severe due to ALS progression. In the late phase, patients become completely dependent on mobility, respiratory issues, dysarthria, and dysphagia. It is important to note that exercise can significantly impact the health and well-being of those with neurodegenerative diseases. After diagnosis, one of the first steps to maximize the functionality of PALS is to prescribe such physical exercises. For PALS, physiotherapy can be customized to their specific requirements and needs. It can help address symptoms and maximize the functionality of PALS, allowing them to live life to the fullest. Commonly advised exercises for PALS include ROM and exercises that mainly involve stretching. To achieve therapeutic goals, strength training for muscles employing low-to-mild intensity and load, coupled with aerobic exercises, for example, stationary cycling, swimming, and walking, are some of the exercises frequently used to treat PALS. In the early and middle phases of ALS, strength training and aerobic exercises are considered more suitable for PALS. Moreover, it is crucial for physiotherapists to strike an equilibrium between underworking and overworking patients and to make accommodations according to their responses and other disease-related factors. PALS should be careful not to push themselves too much and to track any signs of overuse or fatigue in an exercise log that can be reviewed by their physical therapist. If any symptoms do occur, it is important to cease the exercises and re-evaluate with the physiotherapist [6,18,24].

Therapy for Bulbar Symptoms

Management of dysphagia: Bulbar symptoms such as dysarthria and dysphagia are the most typical features of ALS and can dramatically reduce the quality of life. Dysphagia is commonly associated with aspiration, drooling, malnutrition (accompanied by weight loss), and dehydration. PALS patients who display bulbar symptoms experience more severe swallowing complications, such as aspiration. Swallowing dysfunction occurs because of the inefficacy of oral movements, laryngeal elevation, pharyngeal contraction, and restriction of tongue base movements. Early and personalized nutritional care should be provided to PALS in their initial phase of disease due to the weight loss associated with bulbar symptoms such as dysphagia. The loss of body fat and lean mass relates to the decreased energy reserve in PALS. Jejunostomy, or gastrotomy, is an alternative way of feeding PALS. The benefit of gastrotomy is enhanced nutrition, as nutrients come directly to the stomach via this procedure. These disabilities are examined by the use of instrumented methods, such as videofluoroscopy and fibroendoscopic evaluation of swallowing. Nutritional support is indicated in PALS that demonstrate severe problems with swallowing. Percutaneous endoscopic gastrotomy (PEG) tube placement is appropriate for PALS with dysphagia to administer proper medication and nutrition [20,32].

Management of dysarthria*: *Speech loss is a significant challenge for PALS and their loved ones, with dysarthria being the primary symptom in 25%-37% of cases and affecting over 80% of patients as the disease progresses. Effective treatment of dysarthria is crucial for providing multidisciplinary care for ALS. The most commonly used measures in the early stages include speech therapy, and in the advanced stages, communication devices are added. Electronic machines coupled with voice output or head/eye tracking systems can greatly improve communication speed and the character of life for PALS. Patients using communication devices reported little-to-no struggle with handling and found the devices to have a stronger impact on their character of life in comparison to speech therapy. Although speech therapy can be helpful in the initial phase of the disease, it slightly retards the inevitable deterioration in speech, which may explain why patients rate its impact lower. In contrast, communication devices can restore the communicative ability of PALS with advanced dysarthria/anarthria. Communication systems enhance the character of mood and life in individuals with ALS and should be given in the initial stage of the disease. With the use of communication devices, PALS can also cope with their depression and psychological distress. Augmentative and alternative communication (AAC) can meet most of the objectives that are part of the palliative care of PALS. Communication is fundamental for human beings, and using communication devices to give some of these PALS the ability to express themselves again is monumental [21-23,33].

Management of respiratory problems: Chronic respiratory failure is one of the most common and incapacitating features accompanying PALS. Due to weakness in the bulbar, expiratory, and inspiratory muscles, PALS can take advantage of mechanical devices that cater to cough augmentation and ventilatory support. Initial symptoms related to weakness of respiratory muscles greatly vary and mainly involve shortness of breath, confusion, poor concentration, orthopnea, sleepiness in the daytime, fatigue, and morning headaches. Moreover, weakness of the expiratory muscle leads to reduced cough and clear secretion. As the disease advances, respiratory-related issues become more likely, and most PALS will eventually need some form of treatment. Physical therapists must prioritize asking PALS about symptoms of respiratory insufficiency during every clinical visit. Testing for maximum inspiratory pressure, sniff nasal pressure, vital capacity, peak cough expiratory flow, and vital capacity should be conducted at least every three months to ensure proper care. Noninvasive ventilation is the lifelong treatment for respiratory insufficiency in PALS. The use of noninvasive ventilation through a mouthpiece interface or mask can provide ventilatory support in PALS. Mechanically assisted cough can assist with secretion clearing and provide lung volume recruitment. Airway clearance treatment and noninvasive therapies are regarded as standard treatments for PALS with respiratory failure due to neuromuscular degeneration. Furthermore, pharyngeal and laryngeal complications are treated with various measures, such as breathing and voice exercises, compensatory techniques, body positioning, dietary modifications, and the close cooperation of a multidisciplinary team. PT also works to restrengthen the muscles controlling swallowing and can be a useful tool in avoiding pharyngeal complications [18,19,22,34].

Depression and distress management: There is considerable anxiety and depression felt among PALS and their families, along with additional financial and social burdens. Recommended psychological treatment consists of cognitive-behavioral therapy (CBT) supplemented by various coping strategies and situational reappraisal skills. Patients need to be engaged in several healthy and interactive activities to effectively overcome their depression. The objective of CBT is to enhance cognitive and emotional processing in PALS. The character of the life of PALS can be enhanced by improving behavioral patterns through behavioral therapy. Acceptance and commitment therapy (ACT) is predominantly focused on cognitive interventions. The ACT approach includes multiple techniques, such as motivational, acceptance, mindfulness, and behavior change approaches, to eliminate negative thoughts or emotions. These techniques help patients be aware of their present moments and committed actions and improve their psychological flexibility. It is fundamental to take into consideration the heavy care burden that patients' relatives are subjected to, which, in the long term, may affect their health and quality of life. ALS patients with severe disease progression may be offered and have access to palliative care, which can enhance not only patients' quality of life but also family caregivers. Mental health of ALS patients and family caregivers may also be taken into account, and psychotherapy strategies can diminish anxiety and depression and aid with the regrettable outcome of the disease [35-37].

Strengths and limitations

Our narrative review utilized a comprehensive search strategy, including specific keywords and MeSH terms, to gather relevant articles, which increased the likelihood of capturing a wide range of studies. To ensure that the selected articles were aligned with the research topic, explicit inclusion and exclusion criteria were established. The focus on the latest advancements in ALS treatment contributes to the relevance of the review’s findings. However, our review also has some limitations. One potential limitation is excluding non-English studies, which could introduce a language bias and omit valuable research conducted in other languages. Despite involving multiple reviewers, the review is susceptible to reviewer subjectivity during the article screening process. There is also the risk of publication bias, as the review might inadvertently include predominantly positive or significant findings while excluding studies with null or nonsignificant results. Finally, this research paper distinguishes itself through its pioneering approach as one of the few studies in the field of amyotrophic lateral sclerosis treatment. In conclusion, this review not only expands the horizons of research but also holds promise for positively impacting the lives of PALS and shaping the trajectory of future scientific investigations.

## Conclusions

ALS is a severe, debilitating, and ultimately fatal illness despite the tremendous multidisciplinary commitment to seeking a cure and a plethora of treatment studies. The advantages of currently approved therapies are modest. In this review, we looked at ALS and the current treatment modalities that are used in a range of clinical settings. We have identified several plausible factors that may be contributing to the high failure rates present in ALS clinical trials, including but not limited to the selection of the incorrect cellular target, defective clinical trial design, patient selection bias, inadequate medication exposure, lack of compliance, and how uncommon ALS is in the general population, further complicating participation in trials. Although various medications have shown efficacy in animal models of ALS, none have significantly increased longevity or improved the quality of life in PALS. One of the drugs used to treat ALS is *riluzole*, an anti-glutamatergic substance. In recent advances, *edaravone*, an antioxidant and free radical scavenger, has been shown to reduce oxidative stress and delay illness onset. Another licensed drug that improves overall survival and day-to-day functioning is sodium phenylbutyrate/taurursodiol. Other therapeutic strategies, such as posttranslational modification, are also being researched for their potential advantages in ALS treatment. Nonpharmacological therapies, such as robotics, therapy for bulbar symptoms, communication devices, rehabilitation, PT, and stem cell therapy, require increased attention. They are essential in maximizing functional abilities and improving the overall well-being of PALS. One of the significant challenges for PALS is the prevalence of mental burdens, such as fear, anxiety, and depression. Effective psychological therapies included and integrated into palliative care principles can help patients overcome or cope with fear, anxiety, and loneliness and contribute to an improved end-of-life experience for both patients and their families. Researchers are conducting numerous trials to develop a cure for ALS. Further research is urgently needed to identify new therapeutics that go beyond drug repurposing methods to combat ALS effectively. Because ALS is caused by a variety of factors, different agents can be used to target various factors, which could open new doors for treatment. In terms of emerging treatment modalities, gene therapy and cellular therapy have been shown to target multiple pathogenic mechanisms and enhance clinical function and muscle strength. There is a growing need for more clinical trials on different kinase inhibitors that can suppress neuroinflammation and inhibit apoptosis.
